# Paleo‐metagenomics of North American fossil packrat middens: Past biodiversity revealed by ancient DNA

**DOI:** 10.1002/ece3.6082

**Published:** 2020-02-20

**Authors:** Grace Moore, Michael Tessler, Seth W. Cunningham, Julio Betancourt, Robert Harbert

**Affiliations:** ^1^ Smith College Northampton Massachusetts; ^2^ Sackler Institute for Comparative Genomics American Museum of Natural History New York New York; ^3^ USGS Reston Virginia; ^4^ Stonehill College Easton Massachusetts

**Keywords:** ancient DNA, metagenomics, *Neotoma*, paleoecology

## Abstract

Fossil rodent middens are powerful tools in paleoecology. In arid parts of western North America, packrat (*Neotoma* spp.) middens preserve plant and animal remains for tens of thousands of years. Midden contents are so well preserved that fragments of endogenous ancient DNA (aDNA) can be extracted and analyzed across millennia. Here, we explore the use of shotgun metagenomics to study the aDNA obtained from packrat middens up to 32,000 C^14^ years old. Eleven Illumina HiSeq 2500 libraries were successfully sequenced, and between 0.11% and 6.7% of reads were classified using Centrifuge against the NCBI “nt” database. Eukaryotic taxa identified belonged primarily to vascular plants with smaller proportions mapping to ascomycete fungi, arthropods, chordates, and nematodes. Plant taxonomic diversity in the middens is shown to change through time and tracks changes in assemblages determined by morphological examination of the plant remains. Amplicon sequencing of ITS2 and *rbcL* provided minimal data for some middens, but failed at amplifying the highly fragmented DNA present in others. With repeated sampling and deep sequencing, analysis of packrat midden aDNA from well‐preserved midden material can provide highly detailed characterizations of past communities of plants, animals, bacteria, and fungi present as trace DNA fossils. The prospects for gaining more paleoecological insights from aDNA for rodent middens will continue to improve with optimization of laboratory methods, decreasing sequencing costs, and increasing computational power.

## Graphical abstract

Past biodiversity revealed by ancient DNA metagenomics of North American fossil packrat middens. Here, we test shotgun sequencing of ancient DNA to identify the biological components of Late Pleistocene packrat middens. These methods are the first step in establishing robust paleogenomic methods to the analysis of well‐preserved midden materials.

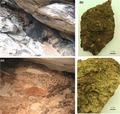

## 1 INTRODUCTION

Steady progress in paleogenetics and paleogenomics over the past few decades is revolutionizing how we study biogeography, evolution, and population dynamics. Much of the progress of ancient DNA (aDNA) has focused on hominins and large mammals, specifically from colder environments at high latitudes and elevations that enhance aDNA preservation (Birks & Birks, 2016; Hagelberg, Hofreiter, & Keyser, 2015). In recent years, much progress has been made on the recovery of aDNA from a variety of substrates including lake, bog, and cave sediments for a range of organisms including bacteria, archaea, plants, and hominins (e.g., Ahmed et al., 2018; Clarke et al., 2019; Parducci et al., 2019; Pedersen et al., 2016; Slon et al., 2017). Various metagenomic and metabarcoding methods have been proposed.

Paleogenomic studies of plants that aim to reconstruct past genetic variation require well‐preserved aDNA from many samples spanning millennia and broad geographic distributions. This is a tall order for most kinds of geologic deposits. One exception are fossil rodent middens, ubiquitous cave deposits that have been well studied across arid parts of North and South Americas and that contain abundant, diverse, and well‐preserved plant macrofossils and other remains (Betancourt & Saavedra, 2002; Betancourt, Devender, & Martin, 1990).

Fossil rodent middens are amalgamations of plant and animal remains embedded in cemented blocks of crystallized urine and preserved for millennia in arid land caves and rock shelters. Because the foraging range of these small rodents is limited, plant macrofossils are assumed to originate from plants growing within 100 m of the midden (Dial & Czaplewsk, 1990). Middens are radiocarbon‐dated from either fecal pellets, multiple plant fragments, or even an individual plant fragment using accelerator mass spectrometry (AMS) dating (Van Devender et al., 1985). Since 1960, >2000 fossil packrat (*Neotoma*) middens have been analyzed and archived from western North America (Strickland, Thompson, & Anderson, 2001). Since 1994, another 1,000 middens produced by other rodents (*Abrocoma*, *Phyllotis*) have been studied from western South America (Latorre, Betancourt, Rylander, & Quade, 2002). Middens provide rich sources of fossil material from both plants and animals, which can be used to investigate ecological responses to environmental change over the last 50,000 years (Balk, Betancourt, & Smith, 2019; Becklin, Medeiros, Sale, & Ward, 2014; Butterfield, Anderson, Holmgren, & Betancourt, 2019; Butterfield, Holmgren, Anderson, & Batancourt, 2019; Dézerald, Latorre, Betancourt, Brito Vera, & González, 2019; Holmgren, Hunter, & Betancourt, 2019).

In past decades, the community of midden researchers has dwindled with few trained in molecular techniques and genomics, delaying full exploration of midden aDNA analysis and its potential from early studies confirming the presence of aDNA in fossil rodent middens. Ancient DNA in middens was used to identify the dung, diet, and Pleistocene biogeography of a rare rodent in northern Chile (Kuch et al., 2002) and of an extinct ground sloth preserved in southern Argentina (Hofreiter, Betancourt, Sbriller, Markgraf, & Gregory McDonald, 2003; Hofreiter et al., 2000). Ancient DNA from a midden in Tiburón Island in the Gulf of California showed that a unique haplotype of desert bighorn sheep inhabited this land‐bridge island 1,500 years ago and that its successful introduction in 1975 represents an “unintentional rewilding” (Wilder et al., 2014). Ancient DNA from middens in the Grand Canyon, USA, provided evidence of papillomavirus (PV) infection and long‐term codivergence with packrats over the last 27,000 years, the oldest known PV sequence (Larsen, Cole, & Worobey, 2018). High‐throughput sequencing and metabarcoding have been reported from South African, Australian (Murray et al., 2012), and Chilean (Díaz et al., 2019) middens. Metabarcoding of middens in the hyperarid Atacama Desert of northern Chile records the response of plant–pathogen communities in response to changing climates during the past 50,000 years (Wood et al., 2018). Because of their dense spatiotemporal distribution, fossil rodent middens in the Americas offer the chance to genetically profile entire communities through time and space and to reconstruct time‐lapse molecular phylogeographies for individual species. To achieve this potential, a necessary step is to continue to improve the genetic characterization of these promising deposits, the principal aim of the present study.

DNA from ancient and highly degraded materials is more accessible than ever before with modern high‐throughput DNA sequencing platforms. Taxonomic composition of ancient samples can be inferred using sophisticated bioinformatic algorithms and massive genetic reference databases (Harbert, 2018). Common sequencing approaches to the taxonomic identification of environmental DNA (eDNA) include amplicon sequencing and whole‐genome shotgun sequencing (Taberlet, Coissac, Pompanon, Brochmann, & Willerslev, 2012). Amplicon sequencing produces short fragments of a specific gene that can be compared to robust databases. In contrast, whole‐genome shotgun sequencing produces random reads from all of the available DNA molecules in a sample, but with a theoretical bias toward high‐copy molecules (e.g., organellar genomes). Amplicon sequencing can sometimes produce imprecise results due to the relatively small samples of the overall genome being compared and because it may produce bias against rare molecules, particularly in degraded samples (Ficetola et al., 2015; Ficetola, Taberlet, & Coissac, 2016; Zinger et al., 2019). Nevertheless, amplicon data may, in some cases, produce more complete taxonomic coverage relative to shotgun methods (Tessler et al., 2017). Whole‐genome shotgun sequencing inherently generates vastly more data from a theoretically larger sample of the genome; therefore, it may overcome underrepresentation of rare molecules and provide more information to make precise taxonomic classifications from highly degraded samples.

For this study, we attempt both shotgun and amplicon methods for metagenomics, but we focus on the whole‐genome shotgun sequencing methods for the exploration of aDNA in packrat midden sequences from western North America. We analyzed aDNA from 25 packrat midden samples of different ages, both unprocessed vouchers (still indurated with crystallized urine) and processed material (after crystallized urine was removed through soaking in water) to determine whether the extraction and classification of endogenous DNA from are possible with both unprocessed and processed material. The packrat middens for which sequencing libraries were attempted in this research are between 300 and 48,000 C^14^ years old and come from two different localities. City of Rocks National Reserve (COR), characterized by mean annual temperature of 9°C and mean annual precipitation of 280 mm, is in south‐central Idaho, USA, near the northern end of the known paleomidden distribution. Guadalupe Canyon on the eastern piedmont of the Sierra Juarez in northern Baja California, Mexico, is characterized by mean annual temperature of 22.5°C and mean annual precipitation of ~70 mm. These two sites more or less span the range of current climatic conditions across which North American packrat middens are preserved and have been studied.

DNA extraction and shotgun sequencing produced data on eleven samples; amplicon samples were reviewed for a subset of six samples. Shotgun metagenomic data were then analyzed to identify taxa present in each sample. The shotgun metagenomic results generally outperform amplicon data and more completely characterize paleo‐communities that are consistent with macrofossil communities identified in the middens and associated taxa.

## 2 MATERIALS AND METHODS

### 2.1 Midden samples

**Figure 1:**
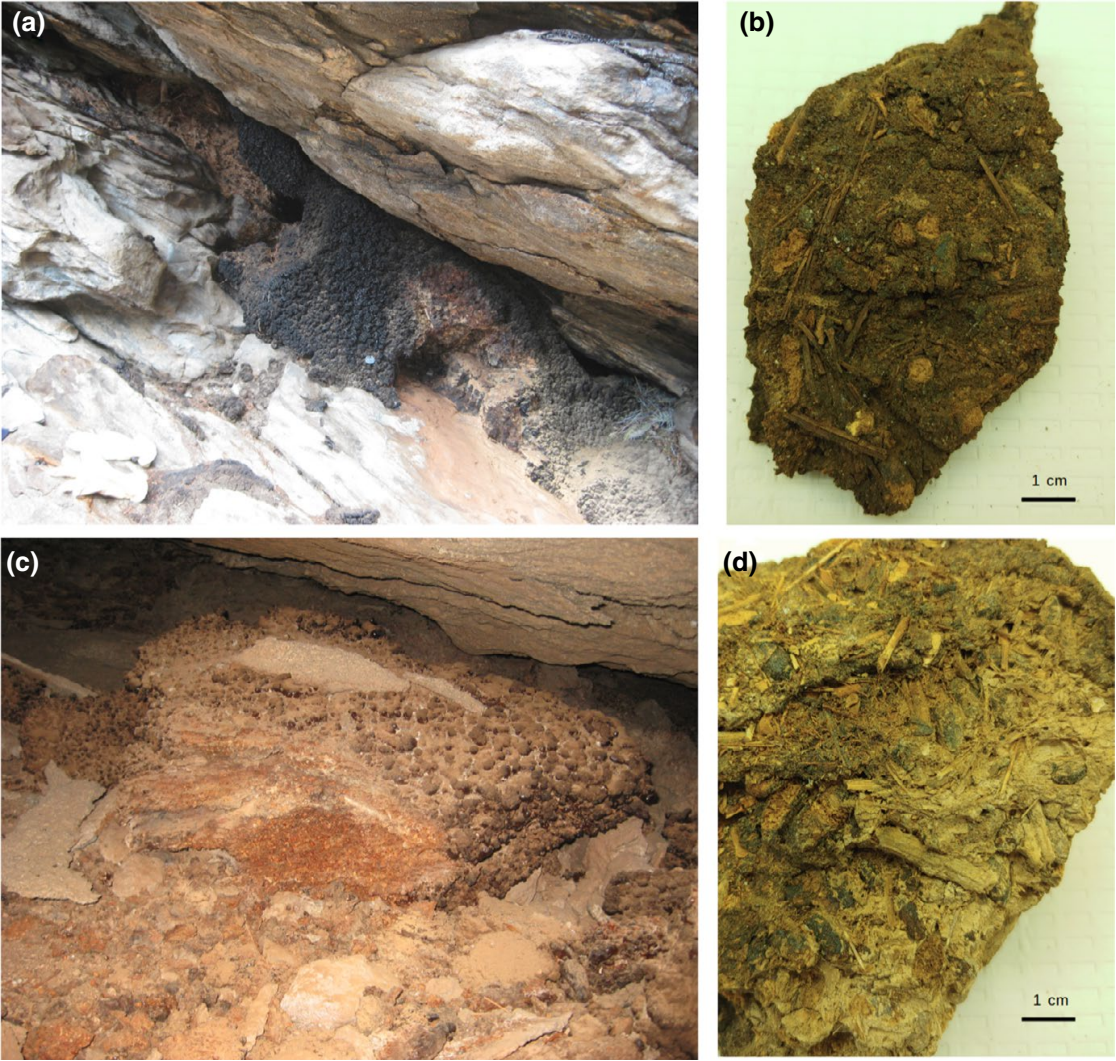
Pleistocene‐age packrat middens from (a,b) City of Rocks National Reserve, south‐central Idaho, USA, and (c,d) Guadalupe Canyon, northeastern Baja California, Mexico

Packrat midden material was sampled from the North American Packrat Midden Collection archived at the University of Arizona's Tree Ring Laboratory, Tucson, AZ. There are two kinds of samples in the Tucson collection, processed and unprocessed (indurated) midden vouchers. Midden processing entails placing a discrete (approximately shoebox‐sized or larger) sample of the midden in a bucket of water. The crystallized packrat urine (amberat; see Figure 1) is soluble in tap water and usually dissolves in one to two weeks, releasing plant material, fecal pellets, and other remains. After dissolving, the soaked midden is wet‐screened through geologic sieves, is dried in a drying oven overnight, and placed into large plastic bags. This material eventually is sorted and identified under a dissecting scope using modern material and then farmed out to other laboratories for geochemical and other analyses. Processed midden is the most abundant material in the University of Arizona Collection. A major question about midden aDNA analysis is the degree to which aDNA survives the current routine processing of middens, which involves both prolonged soaking and oven‐drying the material. Also available for some middens are indurated vouchers, meaning chunks of middens that are still embedded with crystallized urine and have not been processed to release/recover the contained plant macrofossils and other remains. The availability of these indurated vouchers permits assessing of routine processing on midden aDNA.

Both processed and voucher midden samples were taken for DNA extraction from the two sites, twelve from the City of Rocks and thirteen from Guadalupe Canyon. The samples from COR were dated from ~47,500 C^14^ years old to 115 C^14^ years old (initial results partially published in Weppner, Pierce, & Betancourt, 2013). The Guadalupe Canyon samples were dated between 54,400 C^14^ years old and 3,545 C^14^ years old (Holmgren et al., 2014).

### 2.2 DNA extractions

DNA extractions from the middens were completed in an aDNA laboratory at the American Museum of Natural History's Sackler Institute for Comparative Genomics. This is a PCR‐free room with laboratory workspaces designated for low DNA content samples and clean protocols in place to limit residual DNA contamination.

Sampling tools were sterilized in a 20% bleach solution both before starting sampling work and in between each separate midden to remove contaminating bacteria and residual DNA from previous samples and potential modern contaminants. The laboratory bench and all work surfaces (including micropipettes, tube holders, centrifuge, and vortexer) were cleaned with 50% DNA AWAY Surface Decontaminant (Thermo Fisher Scientific). Between 0.25 and 0.8 g were sampled per extraction. Material was scraped off each indurated midden using cleaned and decontaminated forceps, double spatula, and sterile razor blades on new decontaminated workspaces of aluminum foil for each sample. Processed middens were sampled by sorting bulk midden contents into extraction tubes using cleaned and decontaminated forceps.

Extractions were performed using the DNeasy PowerSoil Kit following manufacturers' protocol with the exception that the vortexing disruption step was carried out using a Fisherbrand Bead Mill 24 Homogenizer at 3.10 m/s for 10 min. Final DNA was eluted into 100 μl of 10 mM Tris‐HCl buffer and stored at −20°C. The DNA concentration was quantified using Qubit 2.0 with the dsDNA HS Assay Kit (Life Technologies) as well as an Agilent 2100 Bioanalyzer (Serial No. DE13804219) assay.

### 2.3 Amplicon sequencing and classification

Amplicon sequencing was conducted for a ~185 bp target for *rbcL* [rbcL1 and rbcLB primers (Little, 2014)] and a ~300 bp target for ITS2 [UniPlantF and UniPlantR primers (Moorhouse‐Gann et al., 2018)]. Amplification reactions were set up in a UV‐treated laboratory hood, and PCR was conducted in a laboratory separate from DNA extraction and reaction setup steps. DNA templates were amplified using Fisher Scientific BioReagents Taq DNA Polymerase and Buffer A (500 mM KCl, 15 mM MgCl_2_, and 100 mM Tris‐HCl) in 25 µl reactions with 10 µM primers. PCR cycling conditions for the rbcL amplicon were 40 cycles of 95°C for 30 s, 52°C for 30 s, and 72°C for 30 s with a final extension at 72°C for 10 min. The ITS2 amplification consisted of 40 cycles of 94°C for 45 s, 64°C for 45 s, and 72°C for 45 s with a final extension at 72°C for 10 min. Each sample was amplified in triplicate, and products were cleaned with ExoSAP‐IT. Cleaned products were evenly pooled by DNA concentration for each sample. These products were then sequenced at Genewiz via MiSeq (2 × 250 bp reads).

Using cutadapt (Martin, 2011), the resulting sequences were demultiplexed by locus and had their primer sequences trimmed; furthermore, sequences were only kept if >75 bp. BBMerge (Bushnell, Rood, & Singer, 2017) was used to merge overlapping read pairs, and the resulting full‐length sequences were aligned against the NCBI “nt” database using BLAST (Johnson et al., 2008). Resulting hits (>95% similarity) are reported.

### 2.4 Whole‐genome shotgun sequencing

A total of 21 samples were selected for whole‐genome shotgun sequencing, nine from the Guadalupe Canyon site and 12 from the City of Rocks National Reserve. Samples were chosen for sequencing based on total DNA content (>0.525 ng/µl) and by manual inspection of the Bioanalyzer fragment size distribution to identify samples with majority low molecular weight (<1 kb) DNA. These criteria were imposed under the assumption that endogenous ancient DNA will be highly fragmented and that higher molecular weight DNA is likely from modern contamination. The library preparation was done using the KAPA Hyper Library Preparation Kit (KAPA Biosystems). Library preparation and sequencing were carried out at the New York Genome Center on the Illumina HiSeq 2500 platform for paired‐end reads of 125 base pairs.

### 2.5 Shotgun metagenomic classification

**Figure 2:**
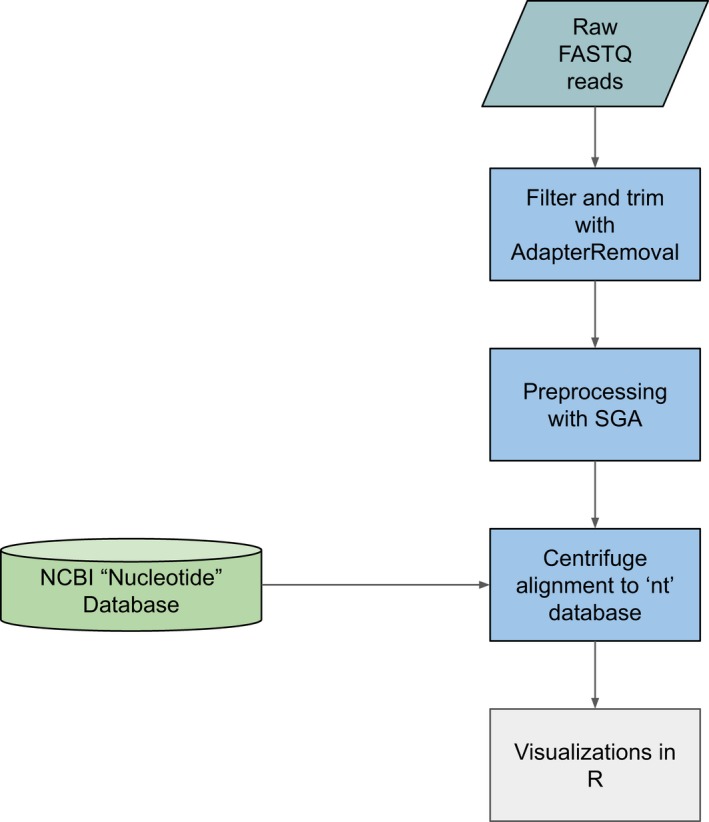
Bioinformatic pipeline for the analysis of the shotgun‐sequenced packrat midden samples. Code, installation instructions, and pipeline settings are available online (https://github.com/rsh249/NeotomaSeq.git)

The whole‐genome shotgun data were processed with a custom pipeline to merge overlapping reads, trim low complexity data, remove duplicate reads and reads shorter than 30 bp, and map reads to the NCBI “nt” reference database (Figure 2). Illumina adapter sequences were removed and overlapping read pairs collapsed using AdapterRemoval (v2.2.2) (Schubert, Lindgreen, & Orlando, 2016) resulting in a sample of the shortest, most likely ancient, molecules. All collapsed reads were next preprocessed by String Graph Assembler (SGA—v0.10.15) (Simpson & Durbin, 2012) to remove reads smaller than 30 bp, low complexity reads, and duplicate sequences. The collapsed and filtered read files were then classified with Centrifuge (v1.0.4) (Kim, Song, Breitwieser, & Salzberg, 2016). Taxonomic assignments were converted to standard classifications based on the NCBI taxonomy database (Federhen, 2012) and the R “taxonomizr” code library (Sherrill‐Mix, 2019). Visualizations of the results were coded in R 3.5.2 (R Core Team, 2019) and the “ggplot2” graphics library (Wickham, 2009).

### 2.6 Analysis of DNA damage

DNA damage and deamination patterns were analyzed using PMDTools (Skoglund et al., 2014) to calculate deamination frequency. All reads were mapped using “*bwa mem*” (Li & Durbin, 2009) to all available chloroplast genomes (ftp://ftp.ncbi.nlm.nih.gov/refseq/release/plastid/). A script for this analysis can be found on the NeotomaSeq Git repository (https://github.com/rsh249/NeotomaSeq/bin/damage_pmdtools.sh).

### 2.7 Shotgun metagenomics pipeline and data availability

All code and parameter settings for individual programs for the shotgun metagenomics portion of this project are published in a public code repository (https://github.com/rsh429/NeotomaSeq), and raw shotgun sequence data are available through the NCBI SRA at BioProject PRJNA488629 (https://www.ncbi.nlm.nih.gov/bioproject/PRJNA488629).

## 3 RESULTS

### 3.1 DNA quantification

**Figure 3:**
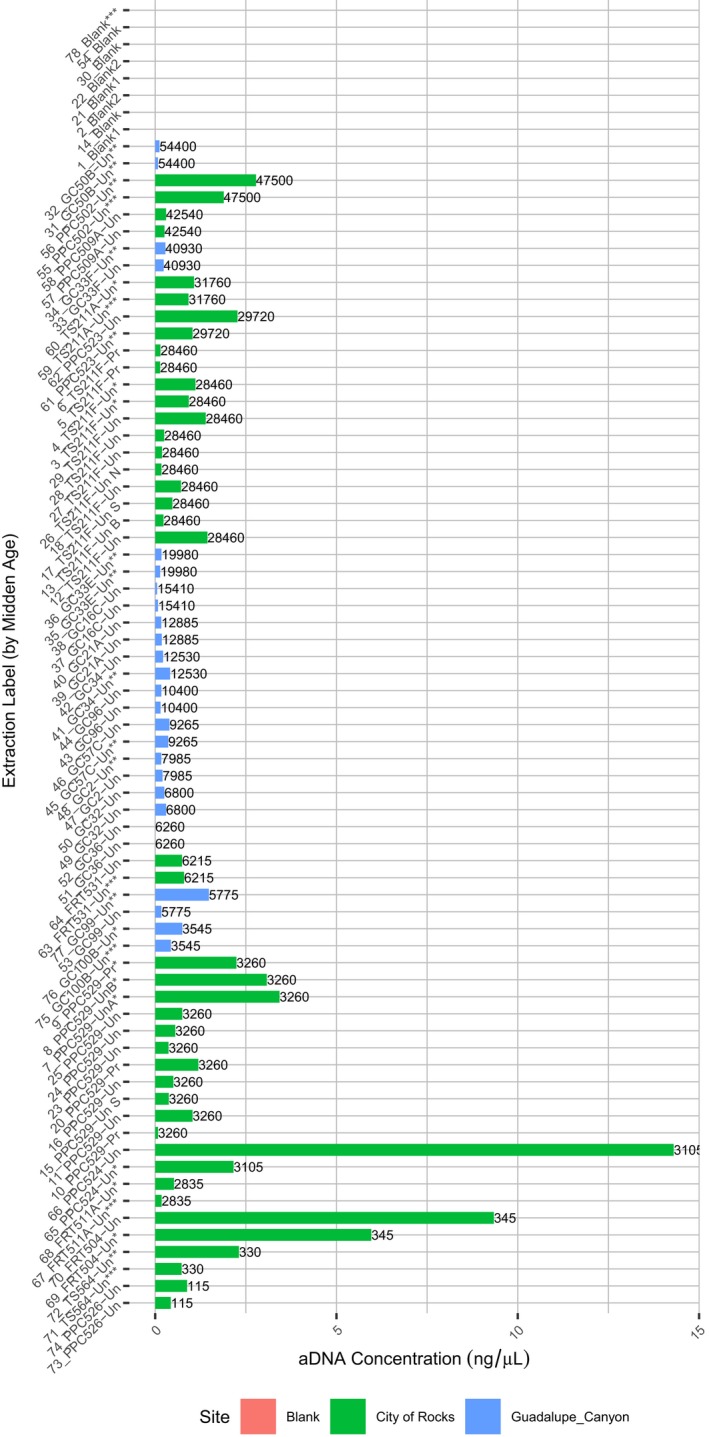
DNA concentration (ng/μl) for midden extractions. Concentrations measured by Qubit 2.0 with the dsDNA HS Assay Kit (Life Technologies) for each of 71 attempted extractions from packrat middens and eight extraction blanks (reagents only). *successfully sequenced samples, **samples that failed library preparation, and ***samples used for amplicon sequencing. Numbers on the end of bars are midden sample ages in radiocarbon years

The concentration of DNA present in each extraction as measured by the 2100 Agilent Bioanalyzer confirms that DNA can be extracted from midden material up to 31,760 years old (Figure 3), but that the yield can be varied unpredictably between and within middens. In a paired *t* test, significantly higher DNA concentrations were achieved from the City of Rocks packrat midden samples (*M* = 1.62 ng/μl, *SD* = 2.60) than the Guadalupe Canyon samples (*M* = 0.267 ng/μl, *SD* = 0.29), *t*(44) = 3.37, *p* = .002. Furthermore, all extraction blanks that were done in parallel with the midden extractions showed undetectable amounts of DNA (Figure 3).

### 3.2 Whole‐genome shotgun sequencing

Eleven of the 21 DNA samples submitted for sequencing were successfully sequenced with midden ages ranging from 345 to 31,760 C^14^ years. The other ten samples failed library preparation procedures and, accordingly, could not be sequenced. Of these ten samples, eight of them were from Guadalupe Canyon, meaning that all but one of the extractions from this hotter, drier site failed to go through library preparation. Overall, between approximately 0.11% and 6.7% of the raw read pairs were uniquely classified given the filtering and classification pipeline (Table 1); however, only processed (washed and sieved) midden samples (sequence samples PPC524‐PrA and TS211F‐2Pr) yielded greater than 1% classified reads.

**Table 1:** Sample data for successful sequencing libraries

Sample name	Sample location	Midden age (C^14^)	DNA concentration (ng/µl)	Raw read count (pairs)	Merged reads	Filtered reads	Total reads classified	Percent reads classified
FRT504‐1‐6‐19	USA	345	5.96	38,984,234	30,242,750	19,887,315	44,794	0.11
FRT511A‐2‐6‐19	USA	2,835	0.516	31,897,277	16,004,936	12,731,946	45,046	0.14
PPC524‐1‐6‐19	USA	3,105	2.16	44,285,310	37,004,021	27,388,646	66,590	0.15
PPC529‐PrA	USA	3,260	2.24	55,738,312	19,207,172	9,196,103	3,766,546	6.7
PPC529‐UnA	USA	3,260	3.43	57,012,411	43,828,129	41,635,079	268,371	0.47
PPC529‐UnB	USA	3,260	3.08	59,879,081	46,247,728	43,967,534	334,688	0.56
TS211F‐2Pr	USA	28,460	4.53	33,887,716	8,294,475	3,580,929	974,043	2.8
TS211F‐4‐6‐7	USA	28,460	1.393	42,884,507	27,683,245	24,182,008	113,778	0.27
TS211F‐Un	USA	28,460	2.036	59,464,854	38,705,809	31,682,218	94,857	0.16
TS211A‐2‐6‐19	USA	31,760	1.07	36,077,665	30,140,241	24,818,818	120,382	0.33
GC100B (Guadalupe Canyon)	Mexico	3,545	0.746	37,080,672	32,584,904	19,363,519	142,956	0.39

### 3.3 Metagenomic classification

**Figure 4:**
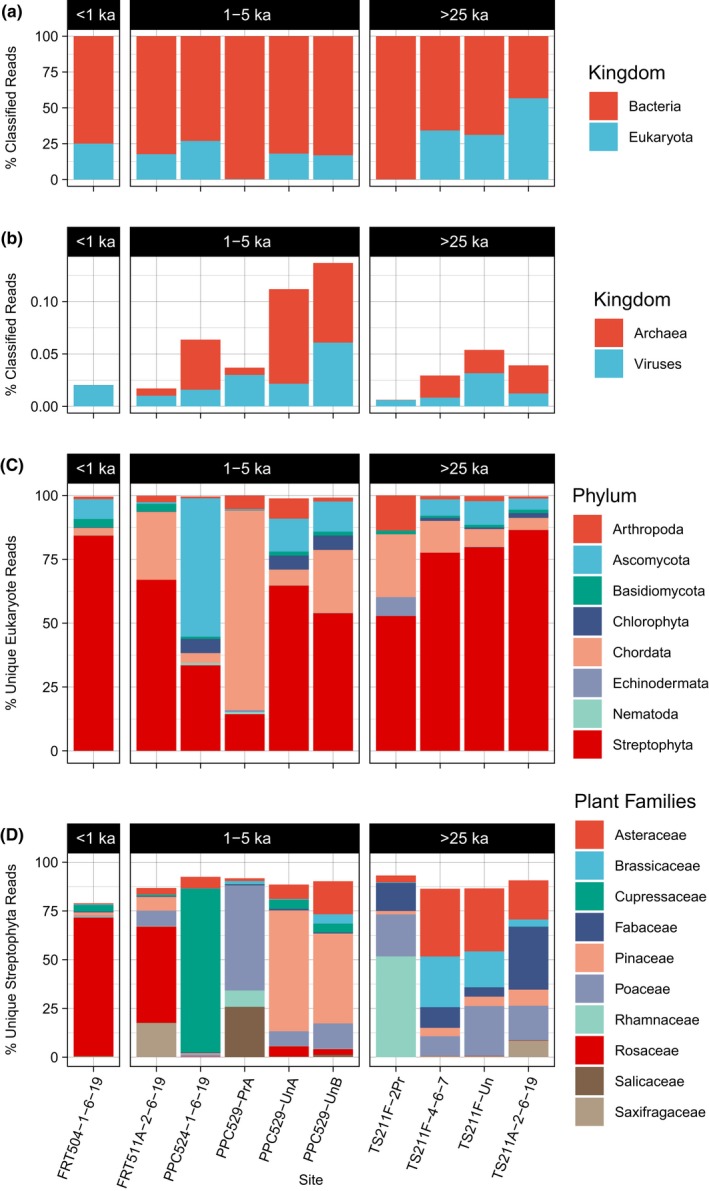
Taxonomic classification of shotgun metagenomic data. (a) Dominant kingdom fractions, (b) minor kingdom fraction (viruses and archaea), (c) breakdown of reads classified as eukaryotes, and (d) breakdown of top ten plant families identified in shotgun metagenomic data. Processed midden samples are indicated with a “Pr” suffix (e.g., PPC529‐PrA); all other middens are indurated voucher specimens

Direct classifications placed some of the DNA fragments at the species level, but there is more confidence in higher classifications (e.g., phylum and family level) where stronger consensus is established. More than half of the classified reads for each sample mapped to bacteria (Figure 4), with Streptophyta, Chordata, and Arthropoda as the dominant eukaryotic phyla. In processed midden samples, nearly all reads were classified as bacteria, whereas in unprocessed (raw, voucher material) midden samples ~25%–50% of classified reads appear to be eukaryotic.

The breakdown of eukaryotic reads by phylum shows that a majority of the reads for each of the COR samples aligned to Streptophyta (Figure 4). Chordata and Arthropoda make up the dominant metazoan phyla, and Ascomycota is the dominant fungal phylum (Figure 4).

As taxonomic classification becomes more specific (e.g., families rather than phyla), greater resolution forms. All of the samples contain some amount of reads aligning to the Pinaceae or Poaceae (Figure 4). The ecosystem seems to shift through a few distinct changes from a sagebrush grassland (dominated by Asteraceae and Poaceae) as seen from the plant composition of middens >25,000 years old to a pinyon–juniper woodland dominated by either Pinaceae or Cupressaceae around 3,000 years ago (Figure 4).

### 3.4 Macrofossil analysis comparison

**Figure 5:**
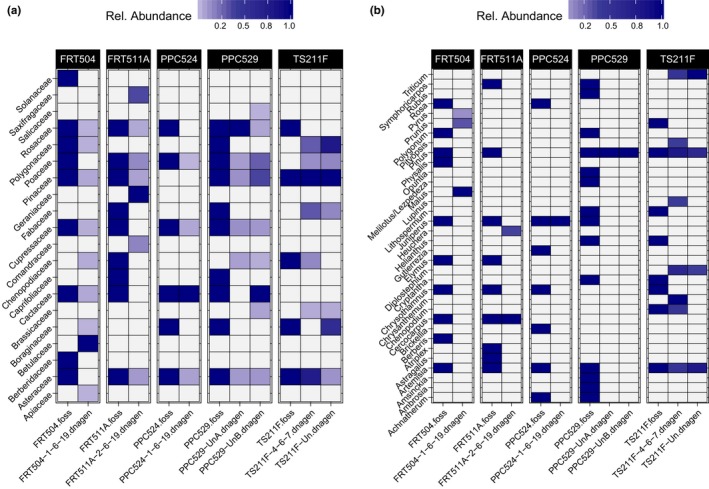
Comparison of plant macrofossil content and DNA classifications. Packrat midden plant macrofossil identifications compared to shotgun metagenomic classifications (>1% of all plant reads) at the (a) family and (b) genus levels. Macrofossil data columns are indicated with the label suffix “.foss.” Metagenomic data columns are indicated by the “.dnagen” label suffix. Taxa shown as identified in the aDNA metagenomic analysis must make up at least 1% of all plant classified reads

Standard plant macrofossil analysis was performed on the middens collected from the City of Rocks locality (Table S1). Plant families identified in the macrofossil analysis are often identified in the aDNA fraction as well (Figure 5a). However, fossil genera and aDNA genera do not often overlap (Figure 5b). The common genera *Artemisia* and *Pinus* do often appear in the aDNA results when also present as macrofossils (Figure 5b). However, for now, we will focus our analysis on aDNA identifications to plant families.

**Figure 6:**
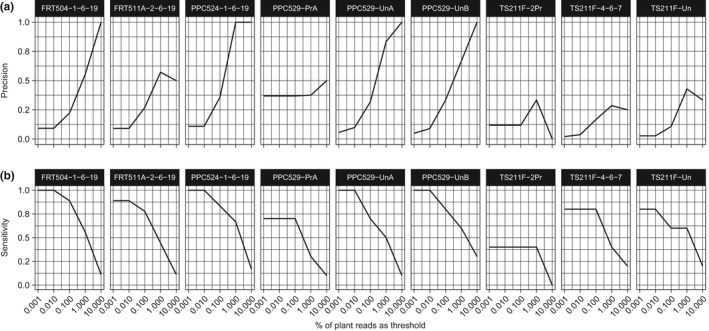
Performance characteristics of metagenomic classification of plant families. (a) Precision [true positives/total fossil], and (b) sensitivity [true positives/(true positives + false positives)] were calculated for the aDNA classifications of plant families using a varying discrimination threshold between 0.001% and 10% of reads classified to plants

The performance of the shotgun metagenomic classifications is assessed here by measuring precision and sensitivity at different thresholds of positive prediction of plant families (% of reads classified). In general, as the threshold for positive prediction increases (from 0.001% to 10% of reads classified) precision increases with fewer false positives, and sensitivity decreases as fewer known macrofossil families are identified in each sample (Figure 6). However, in the processed middens precision and sensitivity are both low (Figure 6).

#### 3.4.1 aDNA Confirmation

**Figure 7:**
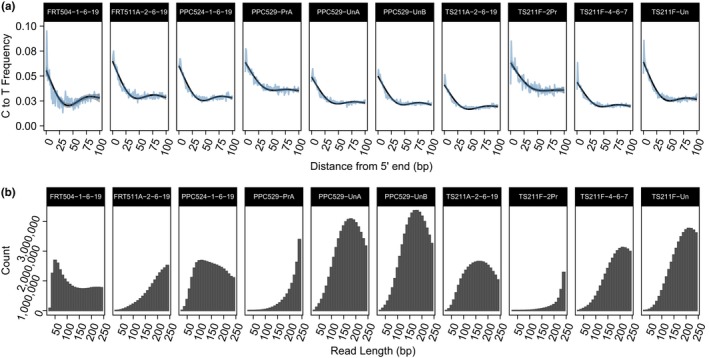
Ancient DNA damage analysis. Cytosine deamination patterns for reads mapping to chloroplast genomes (a) and read length distribution for filtered and merged overlapping paired‐end reads (b) show evidence of ancient plant DNA

Assessment of DNA damage through mapping of cytosine deamination and read length shows that reads that map to chloroplast loci are putatively ancient (Figure 7). In general, all samples analyzed show elevated (5%–10%) cytosine to thymine substitutions near the 5′ end of the DNA molecule dropping to 2.5%–3% by 30bp from the 5′ end (Figure 7a). Also, most samples show read lengths (from merged paired‐end reads) of 50–200 bp (Figure 7b). Sequence reads from the processed midden samples (PPC524‐1‐6‐10 and TS211F‐2Pr) show elevated levels of C to T mismatches across the entire length of the reads relative to the data from raw midden samples (Figure 7a).

## 4 DISCUSSION

Application of metagenomics to packrat midden paleoecology has a lot to contribute to the study of these materials. In a single analysis, aDNA metagenomic data can identify major lineages of eukaryotes—plants, animals, and fungi—and prokaryotes. This method provides the basis for a new way to more rapidly conduct paleoecological studies, as numerous samples can be batch‐processed and data on a wide range of taxonomic groups can be recovered. Also, it does not require specialized knowledge for morphological identification of fragmented macrofossils, helping make studies in this system more available to the scientific community at large.

The taxonomic characterization of DNA from packrat midden series has the potential to generate a millennial‐scale timeline of community composition. These data and future developments to ancient metagenomics methods will aid in understanding the effects of changing environments and provide a clearer picture of the processes involved in ecosystem turnover. This may require improvements to and expansion on the methods presented here, but should be feasible by sampling more ancient packrat middens across a wider transect of geography and time, more replication of sequencing, deeper sequencing, experimentation with target capture and metabarcoding methods, and the establishment of more complete reference databases for global and regional flora and fauna relevant to ecosystems represented in packrat middens.

### 4.1 DNA recovery and midden handling

Processing of packrat middens for macrofossil analysis is not compatible with ancient DNA protocols. In this study, we successfully recover DNA from both processed and voucher specimen middens (Figure 3). Typical analysis of packrat midden plant macrofossils involves dissociating the midden matrix in water (Betancourt et al., 1990). This protocol appears to further degrade any endogenous DNA and introduce significant contamination. The processed samples analyzed here are dominated by bacterial sequences (Figure 4), and the plant families recovered in the metagenomic analysis show low overlap with the plant macrofossil taxa known from those samples (Figure 6). Obvious contaminants include reads mapping to Echinodermata (in one sample) and humans, which indicates the presence of contamination above that of the unprocessed ancient samples (Figure 4). While this is unfortunate, it is not surprising; it has long been known that aDNA samples must be handled with extreme care to preserve the remaining material and to limit contamination from modern sources (Cooper, 2000). However, midden processing is a destructive process in the context of aDNA work, and we encourage developing new field and laboratory protocols that better ensure aDNA preservation, and more routine archiving of unprocessed midden material as voucher specimens.

### 4.2 Macrofossils versus aDNA

In these analyses, we show that DNA extracted from packrat middens can be attributed to a range of organisms consistent with known or likely midden contents (Figure 4). While a large fraction of each sample is bacterial and may represent either endogenous bacteria or potential contaminants, between 20% and 40% of the shotgun metagenomic reads classified are eukaryotic. The majority of these eukaryotic sequences belong to plants (Figure 4). Since plants are the primary midden components, this alone suggests that much of the DNA recovered is endogenous. More convincingly, most of the plant families identified by DNA evidence are also known macrofossil components of the middens analyzed for this study (Figure 5). Although sequences can be identified to the genus level (Figure 5), we do feel that, at this time, the family level is the finest resolution the data should be viewed at due to DNA degradation and lack of available plant genomes for taxa closely related to putative midden components.

When taxa are identified in molecular data but not in the macrofossil record, true aDNA could be present in the form of pollen or other trace material (including unidentifiable macrofossils) that is difficult to access or not typically processed when studying packrat midden contents. The presence of DNA in the absence of pollen or other known tissue is not necessarily surprising and has been observed in other systems, particularly in ancient sedimentary DNA (Clarke, Edwards, Brown et al., 2019; Parducci et al., 2019). It may also be that DNA fragments are being misclassified due to missing data in the reference database or bias toward more complete genomic records (Harbert, 2018). However, the putative ancient sequences described here closely match the appropriate midden surroundings (Figure 6) and importantly are missing common wind‐pollinated genera (e.g., *Quercus* and *Platanus*) that inhabit Central Park, which is the closest pollen source to where the laboratory work was conducted.

Conversely, there are macrofossils identified in the middens that do not show up in the aDNA analysis. There are at least three reasons why this may be: (a) The specific sample taken from the midden might not have the taxon in question; (b) the aDNA that macrofossils might have once contained is no longer viable for analysis and accordingly failed at the extraction or sequencing step; or (c) there may be a lack of genomic data to place sequence reads in the fossil group. Like any fossil, there is presumably a point at which midden contents will be too degraded to recover DNA from, no matter how sensitive sequencing technology becomes, and this process intuitively should occur more rapidly for some species and tissues, and in some midden localities. For example, the best characterized and highest performing midden samples came from the Pinnacle Pass Cave locality, PPC524 and PPC529 (Figure 6).

### 4.3 Tracking plant communities

It is well established that packrat midden plant macrofossils provide insights into changes in plant communities through time. The patterns and relative abundances of plant families identified in the shotgun metagenomic analysis here provide evidence of similar changes (Figure 4) to what has previously been observed in these middens (Weppner et al., 2013). In the City of Rocks midden series, we observe that the oldest middens contain DNA dominated primarily by Poaceae (grasses) and Asteraceae (composites) (Figure 4d). This composition may suggest tree‐less shrubland, much like modern *Artemisia* sagebrush ecosystems. In the middle section of this series (~1–5 ka), the DNA composition shifts to be dominated by Pinaceae, Cupressaceae (junipers), or Rosaceae, a pattern that is more consistent with an open Juniper woodland similar to the modern vegetation of the region (Figure 4d). Accordingly, metagenomics seems like a viable alternative to macrofossils, but, given the incomplete overlap between these methods, adding metagenomics to prior macrofossil work should indeed refine the understanding of sites even further. Like many new molecular methods that had previously required more manual species identifications, the physical and molecular methods are highly synergistic—combining them creates more refined results [e.g., (Parducci et al., 2019; Weiskopf et al., 2018)]. For instance, sometimes, macrofossils in this study were more effective for certain taxa (e.g., Fabaceae; Figure 5), but metagenomics provides insights into other cases (e.g., all bacteria and most metazoan taxa; Figure 4).

### 4.4 Beyond plants

While our focus is on flowering plants and conifers, there are abundant data on a variety of lineages. Prokaryotes are dominant in the dataset, and further work could potentially tease apart any modern contaminants from focal ancient sequences. Viruses are present in low quantities, but recent work in other packrat middens has isolated ancient viral sequences (Larsen et al., 2018). The nonplant eukaryotes, such as vertebrates and arthropods, are even more workable, given that there are clearly defined macrofossils present in packrat middens for other vertebrates and insects (Elias, 1990, 1992; Hall, Van Devender, & Olson, 1988; MacKay & Elias, 1992; Mead, Devender, & Cole, 1983; Van Devender & Mead, 1978). Not only might this be able to establish what animals are generally in the area (e.g., arthropod sequences are the most common), it could also be useful for paleo‐population work on the packrats themselves as well as their diet and parasites.

### 4.5 Amplicon for packrat aDNA

Metagenomic sequencing in this study far outperformed amplicon results; however, our goal with amplicon sequences was to use somewhat longer fragments (~185 bp for *rbcL* and ~300 bp for ITS2) to see if species‐ or genus‐level identification would be possible from the midden aDNA. Toward our goal, when amplicon sequencing was successful, it was highly capable of getting confident genus‐level identifications. Unfortunately, the success rate for amplifying these amplicon loci was low in our samples (Table 2), and only two of the six tested samples yielded identifiable sequences. Future studies should add onto this work by attempting more replicates and comparing shorter amplicon sequences (that are more likely to amplify and may identify sequences at the genus or family level). In either case, it is worth noting that a clear advantage of metagenomics is its ability to get results on multitudes of taxa from microbes to plants and animals all in a single run, unlike the more singular approach taken in amplicon sequencing. The amplicon method has indeed been successful in South American ancient rodent middens (Díaz et al., 2019), and we expect that further refinement of amplicon sequencing methods applied to ancient packrat middens will yield positive results.

**Table 2:** Packrat midden amplicon sequencing

Midden	ID	Age	Amplicon	Taxa	Hits
Blank1	0N	NA	*	**	
TS564‐Un	17	330	*	**	
FRT511A‐2‐6‐19	13	2,835	*	**	
GC100B‐Un	21	3,545	*	**	
GC99‐Un	1	5,775	*	**	
FRT531‐Un	9	6,215	ITS2	*Opuntia* spp*.*	17
				*Sambucus williamsii*	16
				*Sambucus mexicana*	6
				*Nakamurella multipartita*	1
TS211A‐2‐6‐19	5	31,760	ITS2	*Artemisia* spp*.*	210
				*Chrysothamnus* spp*.*	118
				*Boechera* spp*.*	42
				*Erigeron* spp*.*	36
				*Chaenactis* spp*.*	18
				*Descurainia californica*	13
				*Rosa* spp*.*	10
				*Physaria occidentalis*	6
				*Ericameria* spp*.*	5
				*Pinus* spp*.*	4
				*Acamptopappus* spp*.*	3
				*Agrostis* spp*.*	3
				*Primula* spp*.*	2
				*Elymus* spp*.*	1
				*Lupinus* spp*.*	1
				*Muhlenbergia richardsonis*	1
				*Poa* spp*.*	1
			*rbcL*	*Lupinus* spp*.*	2

* Both *rbcL* and ITS2.

** No matches.

### 4.6 Caveats: aDNA confirmation

The packrat midden aDNA samples analyzed in this study contain fragments of DNA from plants and animals that are consistent with what we would expect to and do find through morphological studies in these same packrat middens. As expected, the DNA recovered from packrat middens is highly degraded (Figure 7). The level of DNA damage provides a challenge for building profiles of taxonomic diversity. The damage assessment results suggest that much of the plant DNA extracted from these middens is ancient and derived from macrofossil fragments in each midden. However, further analysis to filter data based on the DNA damage patterns (Skoglund et al., 2014) may be required to see if DNA from modern sources can be removed from this metagenomic sample. Given the DNA damage results (Figure 7), it is still possible that there may be relatively little ancient or a combination of ancient and modern DNA mixed in our samples. Alternatively, all the DNA could be ancient but degraded and fragmented such that the damage patterns are obscured.

### 4.7 Suggestions for additional analyses

The whole‐genome shotgun metagenomic classification results can and should be tested in future studies with other classifiers, such as FALCON and Kraken (Breitwieser, Baker, & Salzberg, 2018; Pratas et al., 2018; Wood & Salzberg, 2014). These software tools use different methods to classify reads and may result in different classifications than those reported here as classifiers are known to produce variable results (Harbert, 2018; McIntyre et al., 2017). Comparing the results of several classifications could create a more comprehensive image of what is present in the sample if certain taxa are identified by all methods.

### 4.8 Future directions

To maximize the potential of packrat midden ancient DNA, future studies should further account for the complexities of the samples. There are many prospects for follow‐up research on aDNA metagenomics. As with all NGS projects, sequencing depth is important for the scale of insights that may be generated, and as sequencers get cheaper and produce more reads, truly deep sequencing likely will become even more feasible. This may be important, as some bacterial studies find that very deep sequencing may be important to detect rare elements of a community (Nicholls, Quick, Tang, & Loman, 2018). Another technical aspect is the quality of reference databases. There are exceedingly few quality nuclear genomes that are currently deposited for plants. However, this is slowly but surely starting to change with rapidly falling sequencing costs and new technologies like long‐read sequencing (Chen et al., 2018; Jung, Winefield, Bombarely, Prentis, & Waterhouse, 2019). As these databases become fuller, projects like this can even simply be reanalyzed to get more refined results. Target capture methods, as have recently been developed for plants (Johnson et al., 2019), may also be viable and help to enrich sequence data for regions where reference data with high taxonomic coverage exist. Confidence in aDNA analysis of packrat middens will increase with sequencing more frequent negative controls and increasing replication, as contamination often results from even the most careful studies.

## 5 CONCLUSION

Metagenomics of packrat midden aDNA has the potential to be a boon for paleoecology, helping to transform the field from one that requires substantial expertise for micro‐ and macrofossil identification to one that benefits additionally from experts working with high‐throughput methods and big data. Here, we show that packrat middens up to ~30,000 years old contain recoverable DNA that is taxonomically consistent with macrofossils found in these deposits. Further investigation into the taxonomic composition of middens with aDNA analysis throughout the region could refine our understanding and the timeline of past climate change (Harbert & Nixon, 2018) and species migration and extinction, and this will better inform the study of the effects of current and future climate change. Deeper sequencing and targeted generation of reference sequences should improve classification precision in future work by providing clearer and more easily verifiable taxonomic classifications for degraded and mixed aDNA samples from packrat middens. The results presented here are an important step toward unlocking the potential of packrat middens as a diverse source of Late Quaternary aDNA and expanding the rich ecological information that can be obtained through the study of packrat midden plant macrofossils.

## CONFLICT OF INTEREST

None Declared.

## AUTHOR CONTRIBUTIONS

R.H. and J.B conceptualized the project. J.B collected the middens. G.M. and R.H conducted the shotgun metagenomic sequencing and analyses. M.T. and S.W.C carried out amplicon metabarcoding sequencing and analyses. G.M., M.T., and R.H. wrote the manuscript. S.W.C. and J.B. helped revise later drafts.

## Data Availability

All code and parameter settings for individual programs for the shotgun metagenomics portion of this project are published in a public code repository (https://github.com/rsh429/NeotomaSeq), and raw shotgun sequence data are archived and available through the NCBI SRA at BioProject PRJNA488629 (https://www.ncbi.nlm.nih.gov/bioproject/PRJNA488629).

## References

[ece36082-bib-0001] Ahmed, E. , Parducci, L. , Unneberg, P. , Ågren, R. , Schenk, F. , Rattray, J. E. , … Wohlfarth, B. (2018). Archaeal community changes in Lateglacial lake sediments: Evidence from ancient DNA. Quaternary Science Reviews, 181, 19–29. 10.1016/j.quascirev.2017.11.037

[ece36082-bib-0002] Balk, M. A. , Betancourt, J. L. , & Smith, F. A. (2019). Investigating (a)symmetry in a small mammal's response to warming and cooling events across western North America over the late Quaternary. Quaternary Research, 92(2), 408–415. 10.1017/qua.2019.13

[ece36082-bib-0003] Becklin, K. M. , Medeiros, J. S. , Sale, K. R. , & Ward, J. K. (2014). Evolutionary history underlies plant physiological responses to global change since the last glacial maximum. Ecology Letters, 17(6), 691–699. 10.1111/ele.12271 24636555 PMC4097002

[ece36082-bib-0004] Betancourt, J. L. , & Saavedra, B. (2002). Paleomadrigueras de roedores, un nuevo método para el estudio del Cuaternario en zonas áridas de Sudamérica. Revista Chilena de Historia Natural, 75(3), 527–546. 10.4067/S0716-078X2002000300005

[ece36082-bib-0005] Betancourt, J. L. , Van Devender, T. R. , & Martin, P. S. (1990). Packrat middens: The last 40,000 years of biotic change. Tucson, AZ: University of Arizona Press. 10.1126/science.250.4983.1021-a 17746928

[ece36082-bib-0006] Birks, H. J. B. , & Birks, H. H. (2016). How have studies of ancient DNA from sediments contributed to the reconstruction of Quaternary floras? The New Phytologist, 209(2), 499–506. 26402315 10.1111/nph.13657

[ece36082-bib-0007] Breitwieser, F. P. , Baker, D. N. , & Salzberg, S. L. (2018). KrakenUniq: Confident and fast metagenomics classification using unique k‐mer counts. Genome Biology, 19, 10.1186/s13059-018-1568-0 PMC6238331 30445993

[ece36082-bib-0008] Bushnell, B. , Rood, J. , & Singer, E. (2017). BBMerge – accurate paired shotgun read merging via overlap. PLoS ONE, 12(10), e0185056 10.1371/journal.pone.0185056 29073143 PMC5657622

[ece36082-bib-0009] Butterfield, B. J. , Anderson, R. S. , Holmgren, C. A. , & Betancourt, J. L. (2019). Extinction debt and delayed colonization have had comparable but unique effects on plant community–climate lags since the Last Glacial Maximum. Global Ecology and Biogeography: A Journal of Macroecology, 3, 343.

[ece36082-bib-0010] Butterfield, B. J. , Holmgren, C. A. , Anderson, R. S. , & Betancourt, J. L. (2019). Life history traits predict colonization and extinction lags of desert plant species since the Last Glacial Maximum. Ecology, 100(10), e02817 10.1002/ecy.2817 31291688

[ece36082-bib-0011] Chen, F. , Dong, W. , Zhang, J. , Guo, X. , Chen, J. , Wang, Z. , … Zhang, L. (2018). The sequenced angiosperm genomes and genome databases. Frontiers in Plant Science, 9, 418 10.3389/fpls.2018.00418 29706973 PMC5909171

[ece36082-bib-0067] Clarke, C. L. , Edwards, M. E. , Brown, A. G. , Gielly, L. , Lammers, Y. , Heintzman, P. D. , … Alsos, I. G. (2019). Holocene floristic diversity and richness in northeast Norway revealed by sedimentary ancient DNA (seda DNA) and pollen. Boreas, 48(2), 299–316. 10.1111/bor.12357

[ece36082-bib-0012] Clarke, C. L. , Edwards, M. E. , Gielly, L. , Ehrich, D. , Hughes, P. D. M. , Morozova, L. M. , … Alsos, I. G. (2019). Persistence of arctic‐alpine flora during 24,000 years of environmental change in the Polar Urals. Scientific Reports, 9(1), 1–11. 10.1038/s41598-019-55989-9 31873100 PMC6927971

[ece36082-bib-0013] Cooper, A. (2000). Ancient DNA: Do it right or not at all. Science, 289, 1139 10.1126/science.289.5482.1139b 10970224

[ece36082-bib-0014] Dézerald, O. , Latorre, C. , Betancourt, J. L. , Brito Vera, G. A. , & González, A. L. (2019). Ecological fidelity and spatiotemporal resolution of arthropod death assemblages from rodent middens in the central Atacama Desert (northern Chile). Quaternary Science Reviews, 210, 15–25. 10.1016/j.quascirev.2019.02.029

[ece36082-bib-0015] Dial, K. P. , & Czaplewsk, N. J. (1990). Do woodrat middens accurately represent the animals' environments and diets? The Woodhouse Mesa Study In Van Devender J. L. B. T. R., & Martin P. S. (Eds.), Packrat middens: The last 40,000 years of biotic change (pp. 43–58). Tucson, AZ: The University of Arizona Press.

[ece36082-bib-0016] Díaz, F. P. , Latorre, C. , Carrasco‐Puga, G. , Wood, J. R. , Wilmshurst, J. M. , Soto, D. C. , … Gutiérrez, R. A. (2019). Multiscale climate change impacts on plant diversity in the Atacama Desert. Global Change Biology, 25(5), 1733–1745. 10.1111/gcb.14583 30706600

[ece36082-bib-0017] Elias, S. A. (1990). Observations on the taphonomy of Late Quaternary insect fossil remains in packrat middens of the Chihuahuan Desert. Palaios, 5, 356 10.2307/3514891

[ece36082-bib-0018] Elias, S. A. (1992). Late quaternary zoogeography of the Chihuahuan Desert insect fauna, based on fossil records from packrat middens. Journal of Biogeography, 19, 285 10.2307/2845452

[ece36082-bib-0019] Federhen, S. (2012). The NCBI Taxonomy database. Nucleic Acids Research, 40, D136–D143. 10.1093/nar/gkr1178 22139910 PMC3245000

[ece36082-bib-0020] Ficetola, G. F. , Pansu, J. , Bonin, A. , Coissac, E. , Giguet‐Covex, C. , De Barba, M. , … Taberlet, P. (2015). Replication levels, false presences and the estimation of the presence/absence from eDNA metabarcoding data. Molecular Ecology Resources, 15(3), 543–556. 10.1111/1755-0998.12338 25327646

[ece36082-bib-0021] Ficetola, G. , Taberlet, P. , & Coissac, E. (2016). How to limit false positives in environmental DNA and metabarcoding? Molecular Ecology Resources, 16(3), 604–607. 10.1111/1755-0998.12508 27062589

[ece36082-bib-0022] Hagelberg, E. , Hofreiter, M. , & Keyser, C. (2015). Introduction. Ancient DNA: The first three decades. Philosophical Transactions of the Royal Society of London. Series B, Biological Sciences, 370(1660), 20130371. 25487324 10.1098/rstb.2013.0371 PMC4275880

[ece36082-bib-0023] Hall, W. E. , Van Devender, T. R. , & Olson, C. A. (1988). Late Quaternary Arthropod remains from Sonoran Desert packrat middens, Southwestern Arizona and Northwestern Sonora. Quaternary Research, 29, 277–293. 10.1016/0033-5894(88)90036-1

[ece36082-bib-0024] Harbert, R. S. (2018). Algorithms and strategies in short‐read shotgun metagenomic reconstruction of plant communities. Applications in Plant Sciences, 6(3), e1034 10.1002/aps3.1034 29732264 PMC5895191

[ece36082-bib-0025] Harbert, R. S. , & Nixon, K. C. (2018). Quantitative late quaternary climate reconstruction from plant macrofossil communities in Western North America. Open Quaternary, 4(1), 8 10.5334/oq.46

[ece36082-bib-0026] Hofreiter, M. , Betancourt, J. L. , Sbriller, A. P. , Markgraf, V. , & Gregory McDonald, H. (2003). Phylogeny, diet, and habitat of an extinct ground sloth from Cuchillo Curá, Neuquén Province, southwest Argentina. Quaternary Research, 59(03), 364–378. 10.1016/S0033-5894(03)00030-9

[ece36082-bib-0027] Hofreiter, M. , Poinar, H. N. , Spaulding, W. G. , Bauer, K. , Martin, P. S. , Possnert, G. , & Pääbo, S. (2000). A molecular analysis of ground sloth diet through the last glaciation. Molecular Ecology, 9(12), 1975–1984. 10.1046/j.1365-294X.2000.01106.x 11123610

[ece36082-bib-0028] Holmgren, C. A. , Betancourt, J. L. , Peñalba, M. C. , Delgadillo, J. , Zuravnsky, K. , Hunter, K. L. , … Weiss, J. L. (2014). Evidence against a Pleistocene desert refugium in the Lower Colorado River Basin. Journal of Biogeography, 41(9), 1769–1780. 10.1111/jbi.12337

[ece36082-bib-0029] Holmgren, C. A. , Hunter, K. L. , & Betancourt, J. L. (2019). Creosote bush (*Larrea tridentata*) ploidy history along its diploid‐tetraploid boundary in southeastern Arizona‐southwestern New Mexico, USA. Journal of Arid Environments, 164, 7–11. 10.1016/j.jaridenv.2019.02.002

[ece36082-bib-0030] Johnson, M. G. , Pokorny, L. , Dodsworth, S. , Botigué, L. R. , Cowan, R. S. , Devault, A. , … Wickett, N. J. (2019). A universal probe set for targeted sequencing of 353 nuclear genes from any flowering plant designed using k‐Medoids clustering. Systematic Biology, 68(4), 594–606. 10.1093/sysbio/syy086 30535394 PMC6568016

[ece36082-bib-0031] Johnson, M. G. , Zaretskaya, I. , Raytselis, Y. , Merezhuk, Y. , McGinnis, S. , & Madden, T. L. (2008). NCBI BLAST: A better web interface. Nucleic Acids Research, 36, W5–W9. 10.1093/nar/gkn201 18440982 PMC2447716

[ece36082-bib-0032] Jung, H. , Winefield, C. , Bombarely, A. , Prentis, P. , & Waterhouse, P. (2019). Tools and strategies for long‐read sequencing and de novo assembly of plant genomes. Trends in Plant Science, 10.1016/j.tplants.2019.05.003 31208890

[ece36082-bib-0033] Kim, D. , Song, L. , Breitwieser, F. P. , & Salzberg, S. L. (2016). Centrifuge: Rapid and sensitive classification of metagenomic sequences. Genome Research, 26(12), 1721–1729. 10.1101/gr.210641.116 27852649 PMC5131823

[ece36082-bib-0034] Kuch, M. , Rohland, N. , Betancourt, J. L. , Latorre, C. , Steppan, S. , & Poinar, H. N. (2002). Molecular analysis of an 11,700‐year‐old rodent midden from the Atacama Desert. Chile. Molecular Ecology, 11(5), 913–924. 11975707 10.1046/j.1365-294x.2002.01492.x

[ece36082-bib-0035] Larsen, B. B. , Cole, K. L. , & Worobey, M. (2018). Ancient DNA provides evidence of 27,000‐year‐old papillomavirus infection and long‐term codivergence with rodents. Virus Evolution, 4(1), vey014 10.1093/ve/vey014 29977605 PMC6007503

[ece36082-bib-0036] Latorre, C. , Betancourt, J. L. , Rylander, K. A. , & Quade, J. (2002). Vegetation invasions into absolute desert: A 45 000 yr rodent midden record from the Calama‐Salar de Atacama basins, northern Chile (lat 22–24 S). Geological Society of America Bulletin, 114(3), 349–366. 10.1130/0016-7606(2002)114<0349:VIIADA>2.0.CO;2

[ece36082-bib-0037] Li, H. , & Durbin, R. (2009). Fast and accurate short read alignment with Burrows‐Wheeler transform. Bioinformatics, 25(14), 1754–1760. 10.1093/bioinformatics/btp324 19451168 PMC2705234

[ece36082-bib-0038] Little, D. P. (2014). A DNA mini‐barcode for land plants. Molecular Ecology Resources, 14(3), 437–446. 10.1111/1755-0998.12194 24286499

[ece36082-bib-0039] MacKay, W. P. , & Elias, S. A. (1992). Late Quaternary ant fossils from packrat middens (Hymenoptera: Formicidae): Implications for climatic change in the Chihuahuan Desert. Psyche: A Journal of Entomology, 99, 169–184. 10.1155/1992/82014

[ece36082-bib-0040] Martin, M. (2011). Cutadapt removes adapter sequences from high‐throughput sequencing reads. EMBnet.journal, 17(1), 10 10.14806/ej.17.1.200

[ece36082-bib-0041] McIntyre, A. B. R. , Ounit, R. , Afshinnekoo, E. , Prill, R. J. , Hénaff, E. , Alexander, N. , … Mason, C. E. (2017). Comprehensive benchmarking and ensemble approaches for metagenomic classifiers. Genome Biology, 18(1), 182 10.1186/s13059-017-1299-7 28934964 PMC5609029

[ece36082-bib-0042] Mead, J. I. , Van Devender, T. R. , & Cole, K. L. (1983). Late Quaternary small mammals from Sonoran Desert packrat middens, Arizona and California. Journal of Mammalogy, 64, 173–180. 10.2307/1380775

[ece36082-bib-0043] Moorhouse‐Gann, R. J. , Dunn, J. C. , de Vere, N. , Goder, M. , Cole, N. , Hipperson, H. , & Symondson, W. O. C. (2018). New universal ITS2 primers for high‐resolution herbivory analyses using DNA metabarcoding in both tropical and temperate zones. Scientific Reports, 8(1), 8542 10.1038/s41598-018-26648-2 29867115 PMC5986805

[ece36082-bib-0044] Murray, D. C. , Pearson, S. G. , Fullagar, R. , Chase, B. M. , Houston, J. , Atchison, J. , … Bunce, M. (2012). High‐throughput sequencing of ancient plant and mammal DNA preserved in herbivore middens. Quaternary Science Reviews, 58, 135–145. 10.1016/j.quascirev.2012.10.021

[ece36082-bib-0045] Nicholls, S. M. , Quick, J. C. , Tang, S. , & Loman, N. J. (2018). Ultra‐deep, long‐read nanopore sequencing of mock microbial community standards. Giga Science, 8(5), giz043. 10.1093/gigascience/giz043 PMC6520541 31089679

[ece36082-bib-0046] Parducci, L. , Alsos, I. G. , Unneberg, P. , Pedersen, M. W. , Han, L. U. , Lammers, Y. , … Wohlfarth, B. (2019). Shotgun environmental DNA, Pollen, and Macrofossil Analysis of Lateglacial Lake Sediments from Southern Sweden. Frontiers in Ecology and Evolution, 7 10.3389/fevo.2019.00189

[ece36082-bib-0047] Pedersen, M. W. , Ruter, A. , Schweger, C. , Friebe, H. , Staff, R. A. , Kjeldsen, K. K. , … Willerslev, E. (2016). Postglacial viability and colonization in North America's ice‐free corridor. Nature, 537(7618), 45 10.1038/nature19085 27509852

[ece36082-bib-0048] Pratas, D. , Pinho, A. J. , Silva, R. M. , João, M. O. , Hosseini, M. , Caetano, T. , & Paulo, J. S. (2018). FALCON‐meta: A method to infer metagenomic composition of ancient DNA. bioRxiv. 10.1101/267179

[ece36082-bib-0049] R Core Team . (2019). R: A language and environment for statistical computing (Version 3.5.2). Retrieved from https://www.R-project.org/

[ece36082-bib-0050] Schubert, M. , Lindgreen, S. , & Orlando, L. (2016). AdapterRemoval v2: Rapid adapter trimming, identification, and read merging. BMC Research Notes, 9, 88 10.1186/s13104-016-1900-2 26868221 PMC4751634

[ece36082-bib-0051] Sherrill‐Mix, S. (2019). taxonomizr: Functions to work with NCBI accessions and taxonomy (Version 0.5.3.). Retrieved from https://CRAN.R-project.org/package=taxonomizr

[ece36082-bib-0052] Simpson, J. T. , & Durbin, R. (2012). Efficient de novo assembly of large genomes using compressed data structures. Genome Research, 22(3), 549–556. 10.1101/gr.126953.111 22156294 PMC3290790

[ece36082-bib-0053] Skoglund, P. , Northoff, B. H. , Shunkov, M. V. , Derevianko, A. P. , Pääbo, S. , Krause, J. , & Jakobsson, M. (2014). Separating endogenous ancient DNA from modern day contamination in a Siberian Neandertal. Proceedings of the National Academy of Sciences, 111(6), 2229–2234. 10.1073/pnas.1318934111 PMC3926038 24469802

[ece36082-bib-0054] Strickland, L. E. , Thompson, R. S. , & Anderson, K. H. (2001). USGS/NOAA North American packrat midden database data dictionary. Reston, VA: US Department of the Interior, US Geological Survey.

[ece36082-bib-0055] Slon, V. , Hopfe, C. , Weiß, C. L. , Mafessoni, F. , De La Rasilla, M. , Lalueza-Fox, C. , … Stewart, J. R. (2017). Neandertal and Denisovan DNA from Pleistocene sediments. Science, 356(6338), 605–608. 28450384 10.1126/science.aam9695

[ece36082-bib-0056] Taberlet, P. , Coissac, E. , Pompanon, F. , Brochmann, C. , & Willerslev, E. (2012). Towards next‐generation biodiversity assessment using DNA metabarcoding. Molecular Ecology, 21(8), 2045–2050. 10.1111/j.1365-294X.2012.05470.x 22486824

[ece36082-bib-0057] Tessler, M. , Neumann, J. S. , Afshinnekoo, E. , Pineda, M. , Hersch, R. , Velho, L. F. M. , … Brugler, M. R. (2017). Large‐scale differences in microbial biodiversity discovery between 16S amplicon and shotgun sequencing. Scientific Reports, 7(1), 6589 10.1038/s41598-017-06665-3 28761145 PMC5537354

[ece36082-bib-0058] Van Devender, T. R. , Martin, P. S. , Thompson, R. S. , Cole, K. L. , Jull, A. J. T. , Long, A. , … Donahue, D. J. (1985). Fossil packrat middens and the tandem accelerator mass spectrometer. Nature, 317(6038), 610–613.

[ece36082-bib-0059] Van Devender, T. R. , & Mead, J. I. (1978). Early Holocene and Late Pleistocene amphibians and reptiles in Sonoran Desert packrat middens. Copeia, 1978, 464 10.2307/1443613

[ece36082-bib-0060] Weiskopf, S. R. , McCarthy, K. P. , Tessler, M. , Rahman, H. A. , McCarthy, J. L. , Hersch, R. , … Siddall, M. E. (2018). Using terrestrial haematophagous leeches to enhance tropical biodiversity monitoring programmes in Bangladesh. Journal of Applied Ecology, 55, 2071–2081. 10.1111/1365-2664.13111

[ece36082-bib-0061] Weppner, K. N. , Pierce, J. L. , & Betancourt, J. L. (2013). Holocene fire occurrence and alluvial responses at the leading edge of pinyon–juniper migration in the Northern Great Basin, USA. Quaternary Research, 80(2), 143–157. 10.1016/j.yqres.2013.06.004

[ece36082-bib-0062] Wickham, H. (2009). ggplot2: Elegant graphics for data analysis. Berlin, Germany: Springer Science & Business Media.

[ece36082-bib-0063] Wilder, B. T. , Betancourt, J. L. , Epps, C. W. , Crowhurst, R. S. , Mead, J. I. , & Ezcurra, E. (2014). Local extinction and unintentional rewilding of bighorn sheep (*Ovis canadensis*) on a desert island. PLoS ONE, 9(3), e91358 10.1371/journal.pone.0091358 24646515 PMC3960132

[ece36082-bib-0064] Wood, D. E. , & Salzberg, S. L. (2014). Kraken: Ultrafast metagenomic sequence classification using exact alignments. Genome Biology, 15(3), R46 10.1186/gb-2014-15-3-r46 24580807 PMC4053813

[ece36082-bib-0065] Wood, J. R. , Díaz, F. P. , Latorre, C. , Wilmshurst, J. M. , Burge, O. R. , & Gutiérrez, R. A. (2018). Plant pathogen responses to Late Pleistocene and Holocene climate change in the central Atacama Desert, Chile. Scientific Reports, 8(1), 17208 10.1038/s41598-018-35299-2 30464240 PMC6249261

[ece36082-bib-0066] Zinger, L. , Bonin, A. , Alsos, I. G. , Bálint, M. , Bik, H. , Boyer, F. , … Taberlet, P. (2019). DNA metabarcoding—Need for robust experimental designs to draw sound ecological conclusions. Molecular Ecology, 28(8), 1857–1862. 10.1111/mec.15060 31033079

